# Analysis of cell free DNA to predict outcome to bevacizumab therapy in colorectal cancer patients

**DOI:** 10.1038/s41525-024-00415-x

**Published:** 2024-05-29

**Authors:** Tom Venken, Ian S. Miller, Ingrid Arijs, Valentina Thomas, Ana Barat, Johannes Betge, Tianzuo Zhan, Timo Gaiser, Matthias P. Ebert, Alice C. O’Farrell, Jochen Prehn, Rut Klinger, Darran P. O’Connor, Brian Moulton, Verena Murphy, Garazi Serna, Paolo G. Nuciforo, Ray McDermott, Brian Bird, Gregory Leonard, Liam Grogan, Anne Horgan, Nadine Schulte, Markus Moehler, Diether Lambrechts, Annette T. Byrne

**Affiliations:** 1 Laboratory for Translational Genetics, Department of Human Genetics, KU Leuven, Leuven, Belgium; 2 VIB Center for Cancer Biology, Leuven, Belgium; 3 Precision Cancer Medicine Group, Department of Physiology and Medical Physics, Royal College of Surgeons in Ireland, Dublin, Ireland; 4 Centre for Systems Medicine, Department of Physiology and Medical Physics, Royal College of Surgeons in Ireland, Dublin, Ireland; 5 Department of Medicine II, University Medical Center Mannheim, Medical Faculty Mannheim, Heidelberg University, Mannheim, Germany; 6 Junior Clinical Cooperation Unit Translational Gastrointestinal Oncology and Preclinical Models, German Cancer Research Center (DKFZ), Heidelberg, Germany; 7 DKFZ-Hector Cancer Institute at University Medical Center Mannheim, Mannheim, Germany; 8 German Cancer Consortium (DKTK), Heidelberg, Germany; 9 Molecular Medicine Partnership Unit, European Molecular Biology Laboratory, Heidelberg, Germany; 10 UCD Conway Institute of Biomolecular and Biomedical Science, University College Dublin, Dublin, Ireland; 11 Department of Pharmacy and Biomolecular Sciences, Royal College of Surgeons in Ireland, Dublin, Ireland; 12 Cancer Trials Ireland, Dublin, Ireland; 13 Val d’Hebron Institute of Oncology, Barcelona, Spain; 14 Department of Medical Oncology, Tallaght University Hospital, Dublin, Ireland; 15 Department of Medical Oncology, St. Vincent’s University Hospital, Dublin, Ireland; 16 Bon Secours Cork Cancer Centre, Bon Secours Hospital Cork, Cork, Ireland; 17 University Hospital Galway, Newcastle Road, Galway, Ireland; 18 Medical Oncology Department, Beaumont Hospital, Dublin, Ireland; 19 Department of Medical Oncology, South East Cancer Center, University Hospital Waterford, Waterford, Ireland; 20 Department of Medicine, Johannes-Gutenberg University Clinic, Mainz, Germany

**Keywords:** Colon cancer, Cancer genomics, Predictive markers

## Abstract

To predict outcome to combination bevacizumab (BVZ) therapy, we employed cell-free DNA (cfDNA) to determine chromosomal instability (CIN), nucleosome footprints (NF) and methylation profiles in metastatic colorectal cancer (mCRC) patients. Low-coverage whole-genome sequencing (LC-WGS) was performed on matched tumor and plasma samples, collected from 74 mCRC patients from the AC-ANGIOPREDICT Phase II trial (NCT01822444), and analysed for CIN and NFs. A validation cohort of plasma samples from the University Medical Center Mannheim (UMM) was similarly profiled. 61 AC-ANGIOPREDICT plasma samples collected before and following BVZ treatment were selected for targeted methylation sequencing. Using cfDNA CIN profiles, AC-ANGIOPREDICT samples were subtyped with 92.3% accuracy into low and high CIN clusters, with good concordance observed between matched plasma and tumor. Improved survival was observed in CIN-high patients. Plasma-based CIN clustering was validated in the UMM cohort. Methylation profiling identified differences in CIN-low vs. CIN high (AUC = 0.87). Moreover, significant methylation score decreases following BVZ was associated with improved outcome (*p* = 0.013). Analysis of CIN, NFs and methylation profiles from cfDNA in plasma samples facilitates stratification into CIN clusters which inform patient response to treatment.

## Introduction

Colorectal cancer (CRC) is the third most frequently diagnosed malignancy in both men and women and represents an important contributor to worldwide cancer mortality and morbidity^1^. Despite best efforts, almost half of patients diagnosed will develop metastatic colorectal cancer (mCRC). Up to 50% of mCRC patients will harbour a RAS mutation^2^ thereby dictating treatment strategies. Current treatment for RAS mutant mCRC includes 5-fluoruracil-based standard-of-care chemotherapy (SOC) combined with the angiogenesis inhibitor bevacizumab (BVZ). Results from Phase III clinical trials have shown that the addition of BVZ to SOC improves response rate and extends survival of mCRC patients^3–5^. Nevertheless, only a subset of patients respond, and overall clinical benefit is limited, with most patients ultimately succumbing^4^. Whilst several novel genomic entities have been proposed as putative BVZ response predictors^6–10^, no robust validated biomarker for BVZ in CRC has emerged. To address this issue we previously studied copy number alterations (CNAs) as potential biomarkers of BVZ response in mCRC^11,12^. Specifically, we classified mCRC tumors into 3 subgroups (cluster 1–3) each characterized by different degrees of CIN. When overlaying treatment response, tumors belonging to intermediate-to-high instability clusters (clusters 2 and 3) demonstrated improved outcome following chemotherapy + BVZ versus chemotherapy alone. In contrast, low instability tumors (cluster 1) derived no further benefit from BVZ. Furthermore, in an effort to understand the biology underpinning differential response to BVZ, gene set enrichment analysis studies were undertaken^12^. Cluster 1 tumors were characterized by a strong immune-activated microenvironment while cluster 2 and 3 tumors were both characterized by angiogenesis and inflammatory response pathways. Cluster 3 tumors displayed less hypoxia and perixosome activation and increased activation of epithelial-to mesenchymal transition pathways when compared cluster 2 tumors. We also assessed the overlap between the consensus molecular subtype (CMS) signature and the CIN clusters. Cluster 3 was characterized by more CMS4 tumors compared to cluster 2, suggesting that Cluster 3 had higher levels of CIN and fewer hypermutated tumors than cluster 2. In summary, we have previously identified copy number load as a novel predictive biomarker of BVZ response in mCRC^12,13^.

As cancer-specific genetic and epigenetic abnormalities have increasingly been identified through liquid biopsy^13–17^, herein, we sought to develop a minimally invasive, clinically relevant assay to stratify patients into CIN classifiers. cfDNA has recently been employed to monitor tumor response to neoadjuvant and postoperative therapy in patients with metastatic disease^13,16,18,19^. Moreover, we previously demonstrated the utility of detecting CNAs using cfDNA-based low coverage whole-genome sequencing (LC-WGS) analysis for the early detection of ovarian cancer^20^. In addition to WGS read-outs based on CNA, cfDNA can also be leveraged to study nucleosome footprinting (NF) and methylation changes^21^. When cfDNA is released into blood, specific fragmentation patterns can be detected in LC-WGS data using NF. Specifically, differences in cfDNA fragment lengths can provide precise information about cell type, gene expression, cell physiology or pathology. Moreover, NF can enhance the detection sensitivity of circulating tumor DNA (ctDNA)^22^ based on cfDNA fragmentation patterns, as previously demonstrated by NF analysis of ovarian cancer patients^21^.

cfDNA from cancer patients also differs from that of healthy controls with respect to DNA methylation patterns, manifesting global hypomethylation and focal hypermethylation at tumor suppressor genes. Moreover, it has been reported that methylation levels of cfDNA in plasma are consistent with those in the primary tumor^23^. We previously studied methylation profiles to investigate biological mechanisms underpinning therapy response to anti-angiogenics. Specifically, we showed that DNA hypermethylation can serve as a broadly applicable biomarker for tumor hypoxia, which is a key driver for the development of resistance against anti-angiogenic therapies^24^. Mouse tumor models suggested that tumor hypoxia triggers DNA hypermethylation of tumor-suppressor genes in untreated cancer cells, while effective BVZ treatment would restore tumor hypoxia levels and reduce DNA methylation levels. Together, these findings suggest that the cfDNA methylation signature could serve as a prognostic biomarker for minimally invasive cancer therapy monitoring^6^.

In the current study we have developed a prototype liquid biopsy assay for subtyping mCRC patients based on CIN profiles derived from cfDNA LC-WGS data. Firstly, we have shown that analysis of previously established CIN clusters in plasma cfDNA may be used to predict outcome to BVZ in mCRC patients. While NFs established for AC-ANGIOPREDICT samples did not differ between CIN clusters, they nevertheless correlated with cfDNA tumor fractions. We further assessed discrete methylation patterns of CIN clusters in cfDNA and established a prototype on-treatment prognostic BVZ assay which directly tests the hypothesis of blood vessel normalization by anti-angiogenics.

## Results

### Concordance of CIN patterns between matched plasma and tumor samples

**Fig. 1:**
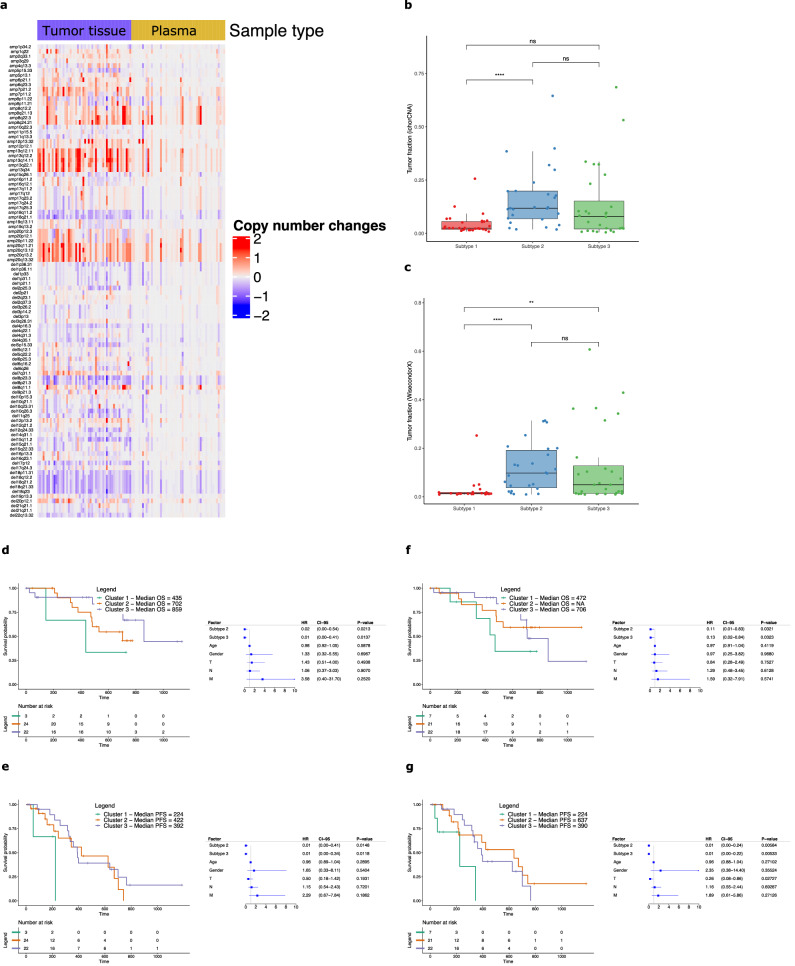
Analysis of CIN in mCRC tumor tissue and plasma samples. **a** Overview of significant copy number aberrations across tumor tissue and plasma samples. Recurrent copy number changes from GISTIC for each sample (*x*-axis) were plotted with respect to the amplifications and deletions (*y*-axis), after calculating copy number aberrations with ASCAT without normalization between tumor tissue and plasma samples. **b**, **c** Tumor fractions were estimated for the AC-ANGIOPREDICT plasma samples using ichorCNA, and WisecondorX. The significant differences are depicted using the following *P* values: *P* < 0.05 (*), *P* < 0.01 (**), *P* < 0.001 (***) and *P* < 0.0001 (****). **d**–**g** Kaplan–Meier plots and multivariate Cox regression of overall survival (OS) or progression free survival (PFS) with hazard ratios, 95% confidence intervals and *P* values for each cluster while correcting for relevant covariates across AC-ANGIOPREDICT tumor tissue or plasma samples. Cluster 1 is considered a reference. Only paired tumor tissue/plasma samples from the AC-ANGIOPREDICT cohort were used.

LC-WGS copy number profiles of 52 plasma samples and matched tumor tissue (see Methods for description of sample characteristics and treatment regimens) from the AC-ANGIOPREDICT mCRC patients (Table 1) who received BVZ were compared to evaluate concordance. We assessed the 43 copy number amplifications and 59 deletions reported previously^12^ to determine CIN subcluster identity. We confirmed the presence of these CNA in various tumor tissue samples, however not all CNAs were retrieved in the corresponding plasma samples due to dilution of the tumor cells by non-cancerous cells (Fig. 1a). Furthermore, we calculated the Spearman correlations of the CNA amplification and deletion peak copy number values derived from the LC-WGS profiles. Patients were ordered by alternating tumor tissue and matching plasma samples (Supplementary Fig. 1). The highest correlation was detected close to the diagonal, indicating a high correlation between tumor tissue and matching plasma of the same patient. Nevertheless, in patient plasma samples that had high sCNA levels, similar sCNA regions in the profile were found to correlate with multiple, unmatched tumor tissue samples

**Table 1:** Patient sample overview from the AC-ANGIOPREDICT (AC-ANGIOPREDICT) cohort (*n* = 74 plasma samples, NCT01822444) and University of Mannheim (UMM) cohort (*n* = 24 plasma samples) that were used for each analysis

Sample overview				
	AC-ANGIOPREDICT		UMM	
	*n* = 74 patients	%	*n* = 24 patients	%
CNA subtyping	52 paired tumor tissue/plasma samples	70	24 (plasma only)	100
Nucleosome scoring	74 plasma samples	100	0	0
Methylation analysis	61 (40 after quality filtering) plasma samples before (baseline) and 61 (40 after quality filtering) plasma samples after treatment with BVZ (week 6)	82 (54 after quality filtering)	0	0

We next classified each plasma sample according to the CIN classifier, determined previously in tumor tissue^12^. Most of the LC-WGS plasma samples (50%) were originally categorized as Cluster 1 (low CIN), which is much more frequent than the expected number of low CIN tumors (11%) we reported previously^12^. However, given the low plasma tumor burden, it is possible that CNAs in plasma samples were underestimated with respect to corresponding tumor tissue samples. We therefore optimized our CNA subtyping approach by adjusting tumor content in each cfDNA sample. Here, we employed a scaling procedure to adjust the CNA profiles. As the tumor fraction (tumor cfDNA) was significantly lower in the plasma of mCRC patients, this procedure was necessary to ensure that the tumor tissue and plasma cfDNA CNA were profiles similar. Therefore, we adjusted the plasma profiles for tumor content. To avoid algorithm bias, we have presented the results from multiple CNA calling methods (i.e. ASCAT, ichorCNA and WisecondorX), to determine copy number profiles from low-coverage whole-sequencing data. Finally, the grouping of cluster 2 and 3 into one cluster (CIN high) is justified since both clusters are comparable in regard to response to BVZ. Following normalization, significantly more CNAs were determined across all samples and results of all tools used to correct for tumor content (see Supplementary Information) are shown in Table 2.

**Table 2:** Overview of the classification results of the 52 AC-ANGIOPREDICT paired tumor tissue and plasma samples with respect to each bioinformatics software tool

Method	Match	nNo match	Ccorrect classification			Incorrect classification					
			P1-T1	P2-T2	P3-T3	P1-T2	P1-T3	P2-T3	P2-T1	P3-T1	P3-T2
ASCAT	16	36	9	3	4	8	9	8	4	5	2
ichorCNA	26	26	2	10	14	6	9	4	3	0	4
WisecondorX	37	15	3	15	19	4	0	6	0	0	5

CIN subtypes in plasma are classified as P1 (subtype 1), P2 (subtype 2) or P3 (subtype 3), while mCRC subtypes of matching tumor tissue are designated as T1 (subtype 1), T2 (subtype 2) or T3 (subtype 3).

Firstly, using ASCAT^25^, we observed that just 16 out of 52 paired samples (30.8%) had an equal cluster classification in plasma and tumor tissue. Notably, several tumor tissue samples in cluster 3 (high CIN) were incorrectly classified as cluster 1 in plasma (*n* = 9). Using ichorCNA^26^, agreement of the paired samples increased to 26 out of 52 samples (50%). Best agreement was established, however, using WisecondorX^27^, with 37 matching samples out of 52 (71%) being assigned to the same cluster. These data indicate that WisecondorX clustering to generate copy number profiles in plasma is the most effective strategy, likely due to the improved normalization of CNV profiles with respect to healthy controls. Supplementary Fig. 2 illustrates examples of the WisecondorX results of paired tumor tissue and plasma samples. Example 1 shows agreement in the copy number profiles between a matched tumor tissue and plasma sample, which are both assigned to cluster 2. The estimated tumor fractions of tumor tissue and plasma were 39·1% and 25·4%, respectively. Example 2 also demonstrates agreement for cluster 3 samples, with estimated tumor fractions of 27·2% and 31·5% for tumor tissue and plasma, respectively.

Nevertheless, some plasma samples were still incorrectly classified using WisecondorX (see Table 2). We identified 4 out of 52 plasma samples (7·7%) that were classified into cluster 1 while the paired tumor tissue was classified into cluster 2. Example 3 in Supplementary Fig. 2 demonstrates such misclassification, with a tumor fraction of 9.9% in tumor tissue but only 1.8% in plasma. While several CNAs were detected in tumor tissue, the tumor fraction in plasma was too low to accurately assign the plasma sample into cluster 2. In addition, given that the CNV profiles of cluster 2 and cluster 3 in the classifier are relatively similar by definition, we found 6 out of 52 plasma samples (11·5%) classified into cluster 2 with paired tumor tissue being cluster 3, and 5 out of 52 plasma samples (9·6%) classified into cluster 3 with paired tumor tissue being cluster 2. However, both clusters 2 and 3 manifest with high CIN levels and therefore these misclassifications are unlikely to influence response prediction to BVZ^12^. In conclusion, after refining the bioinformatics pipeline using WisecondorX we were able to correctly cluster 15 additional matching samples compared to using ASCAT. Considering that 7·7% of the plasma samples were incorrectly classified into cluster 1, we obtain an accuracy of 92·3% to distinguish high CNA (cluster 2 and 3) from low CNA (cluster 1) samples in plasma.

For validation of CIN clustering in plasma, subtypes were defined on a second cohort of plasma-derived cfDNA samples from n = 24 mCRC patients who received BVZ ± SOC chemotherapy designated as UMM cohort (Table 1). Since no paired tumor tissue samples were available in this cohort, the mean ratio between the calculated tumor content in plasma and tumor tissue from the AC-ANGIOPREDICT cohort was used as a scaling factor. Nine samples were found to be classified into cluster 1, 7 into cluster 2 and 9 into cluster 3 (Table 3. Although no paired tumor tissue was available, our pipeline with WisecondorX still managed to classify the expected number of samples in the clusters with high CNA levels (cluster 2 and cluster 3).

**Table 3:** Classification results of the UMM plasma sample cohort, before and after scaling of the copy number profiles, with CIN subtypes shown as P1 (subtype 1), P2 (subtype 2) and P3 (subtype 3)

Method	Classification		
	P1	P2	P3
WisecondorX (unscaled)	13	5	7
WisecondorX (scaled)	9	7	9

Next, tumor fraction was calculated for all plasma samples in the AC-ANGIOPREDICT cohort using ichorCNA (Fig. 1b) and WisecondorX (Fig. 1c). The tumor fraction as estimated by both tools was similar. The WisecondorX tumor fractions of cluster 1 samples were found to be significantly lower compared to cluster 2 and cluster 3 samples. However, no significant tumor fraction difference between cluster 2 and cluster 3 was found. Given the significant differences between the cluster tumor fractions, these results confirm that correcting for tumor fraction is required to improve CNA classification of mCRC plasma samples, as highlighted above.

### Predictive effects of plasma CIN profiles for BVZ in mCRC

Survival within defined clusters of paired samples from the AC-ANGIOPREDICT cohort were assessed in either tumor tissue or plasma (*n* = 52 samples, see Table 2).

PFS in the AC-ANGIOPREDICT and University of Mannheim (UMM) cohort was defined as the time from start of BVZ therapy to progressive disease or death from any cause (whichever occurred first). For tumor tissue samples, multivariate Cox regression confirmed that both cluster 2 and cluster 3 patients correlated with improved overall survival (Fig. 1d) and progression free survival (Fig. 1e). For overall survival, the hazard ratio’s (HR) for cluster 2 and 3 relative to cluster 1 were 0.02 (confidence intervals (CI) 0.00–0.54, *p*-value = 0.0213, Cox regression) and 0.01 (CI 0.00–0.41, *p*-value = 0.0137, Cox regression), respectively. For progression free survival, the HRs for cluster 2 and 3 relative to cluster 1 were 0.01 (CI 0.00–0.41, *p*-value = 0.0148, Cox regression) and 0.01 (CI 0.00–0.34, *p*-value = 0.0118, Cox regression), respectively. Thus, data from the prospectively-collected phase II AC-ANGIOPREDICT trial samples corroborate previous findings from the retrospective cohort^12^. The same effect on overall survival (HR = 0.11, CI 0.01-0.83, *p*-value = 0.0321 for cluster 2 and HR = 0.13, CI 0.02–0.84, *p*-value = 0.0323 for cluster 3) and a stronger effect on progression free survival (HR = 0.01, CI 0.00–0.24, *p*-value = 0.00584 for cluster 2 and HR = 0.01, CI 0.00-0.22, *p*-value = 0.00533 for cluster 3) was observed for CIN subclusters determined on plasma samples, with an improved response to BVZ in CIN-high (namely cluster 2 and 3) patients (Fig. 1f, g).

Overall, while not all plasma samples were assigned to the same cluster as the corresponding tumor tissue samples, the cluster definition of the plasma samples confirmed that CIN-high mCRC tumors demonstrated improved survival compared to CIN-low tumors under BVZ.

### CIN clusters do not manifest distinct nucleosome patterns

**Fig. 2:**
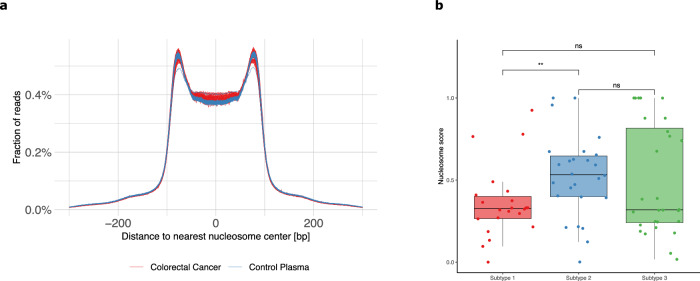
Nucleosome footprint in LC-WGS data. **a** An M-shaped profile is found when a genome-wide distribution of the distances between the start of each read and the center of the nearest nucleosome is plotted. AC-ANGIOPREDICT mCRC plasma samples are shown in blue lines while healthy control samples are shown in red lines. **b** Boxplots of the nucleosome scores of mCRC subtypes. The significant differences are depicted using the following *P*-values: *P* < 0.05 (*), *P* < 0.01 (**), *P* < 0.001 (***) and *P* < 0.0001 (****).

Fragmentation patterns of plasma-derived cfDNA are known to reflect nucleosome positions of cell types contributing to cfDNA^15^. Since a substantial fraction of cfDNA from cancer patients originates from tumor cells, the cfDNA NF differs between cancer patients and healthy controls^28^. Here, the AC-ANGIOPREDICT cohort (*n* = 74, Table 2) was assessed to determine whether NFs could also be used to distinguish mCRC clusters. When plotting the fraction of reads with respect to the distance of each read to the nearest nucleosome center, an M-shaped profile was obtained (Fig. 2a). mCRC plasma samples were observed to be relatively enriched in the center of nucleosomes, while the edges were relatively depleted compared to healthy individuals. Furthermore, when assessing cluster 1 samples only (Supplementary Fig. 3), we observed an enrichment in the center of nucleosomes when compared to healthy control samples. This result confirms that cluster 1 samples represent tumors with low copy number burden (i.e. these results do not merely reflect low tumor purity in these samples).

The degree of nucleosome position deviation in mCRC plasma samples was quantified with respect to reference healthy profiles, whereby a score of 0 corresponds to the healthy profile and a score of 1 corresponds to a mCRC profile. A significant difference (*p-*value = 0.009, Mann-Whitney test) was observed in the nucleosome scores between cluster 1 and cluster 2 samples (Fig. 2b). In contrast, cluster 3 samples displayed a heterogeneous profile, containing both samples with a low and high nucleosome score. As a result, the nucleosome score profile of cluster 3 samples was not significantly different from cluster 1 (*p*-value = 0.491, Mann–Whitney test) and cluster 2 samples (*p*-value = 0.542, Mann-Whitney test). Although it is possible that cluster 1 cfDNA samples consist of different cell types compared to cluster 2 and 3 cfDNA samples, it is more likely that these differences in nucleosome positions correlate with the tumor fraction in these samples. Indeed, we observed strong pairwise correlations between tumor fractions estimated with WisecondorX and nucleosome scores (Spearman’s rho = 0.80, *p*-value < 0.001), confirming that our nucleosome score reflects the tumor fraction in cfDNA samples.

In addition, we investigated if the combination of NF and CIN data would support improved classification accuracy. Using WisecondorX on the 52 paired samples, we found that CIN was able to classify 37 matching samples out of 52 (71%). When comparing high CNA clusters (cluster 2 and 3) with the low CNA cluster (cluster 1), CIN profiling resulted in an AUC of 0.79. In comparison, using NF for the classification only resulted in an AUC of 0.44 for distinguishing cluster 1 from cluster 2 and 3 samples, possibly owing to the heterogeneous profile of cluster 3 samples shown in Fig. 2b. CIN clusters did not manifest distinct nucleosome patterns. Therefore we did not obtain an improved classification accuracy when combining CIN and NF together (AUC of a CIN/NF combined score = 0.76, down from AUC = 0.79 using CIN alone).

### CIN clusters manifest distinct methylation patterns

**Fig. 3:**
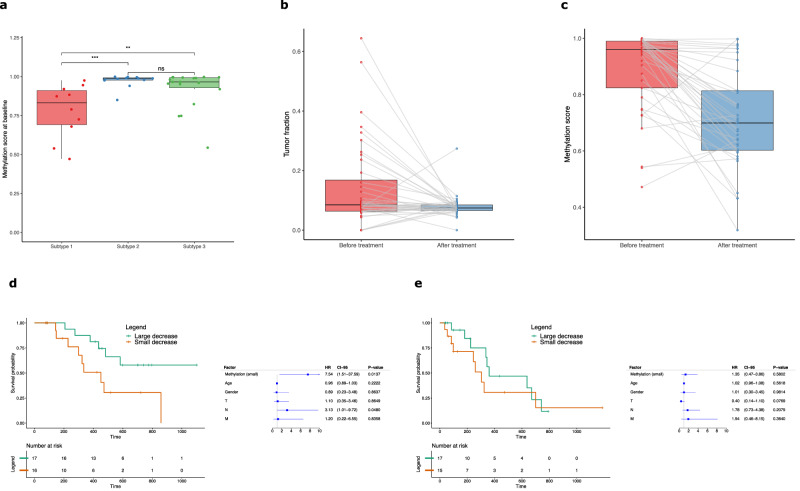
Analysis of targeted methylation profiles. **a** Boxplots of methylation score differences at baseline between mCRC clusters. The significant differences are depicted using the following *P* values: *P* < 0.05 (*), *P* < 0.01 (**), *P* < 0.001 (***) and *P* < 0.0001 (****). **b** Comparison of the calculated tumor fractions of the mCRC targeted methylation samples before and after treatment with BVZ. The tumor fractions were determined using ichorCNA from off-target methylation bisulphite sequencing reads. Samples shared by the same patient are connected by grey lines. **c** Methylation scores of AC-ANGIOPREDICT mCRC samples before and after treatment with BVZ. Samples shared by the same patient are connected by grey lines. **d** Kaplan–Meier plots and multivariate Cox regression of overall survival (OS) with hazard ratios, 95% confidence intervals (CI-95) and *P* values for methylation cluster while correcting for relevant covariates across all samples. Methylation cluster “Large decrease” is considered a reference. **e** Kaplan–Meier plots and multivariate Cox regression of progressive free survival (PFS) with hazard ratios, 95% confidence intervals (CI-95) and *P* values for methylation cluster while correcting for relevant covariates across all samples. Methylation cluster “Large decrease” is considered a reference.

Subtype differences in methylation profiles were also assessed. Targeted bisulphite sequencing was performed on 61 AC-ANGIOPREDICT mCRC plasma samples before treatment and on-treatment (week 6) with BVZ (Table 1), as well as on 41 healthy controls. We first assessed the methylation scores before treatment (Fig. 3a), with samples from cluster 1 demonstrating significantly lower methylation scores compared to cluster 2 and 3 (*p*-value = 0.000619, Mann-Whitney test). No significant difference between cluster 2 and 3 was found (*p*-value = 0.235, Mann-Whitney test). There was no correlation between tumor content and the pre-treatment methylation score (Spearman’s rho = 0.29, *p*-value = 0.083).

In addition to CIN clustering, we investigated whether the methylation score from the methylation data and the nucleosome score from LC-WGS can be used for subtype characterization of AC-ANGIOPREDICT plasma samples (*n* = 40) which were collected pre-treatment. Supplementary Fig. 4 illustrates that in plasma, the methylation score is a more effective metric to distinguish cluster 1 from cluster 2 and 3 samples (AUC = 0.87), compared to the nucleosome score (AUC = 0.75) in this cohort. The efficiency of the methylation score for distinguishing cluster from cluster 2 and 3 samples is comparable to CNA profiling of cfDNA, as we previously found an accuracy of 92·3% using 52 plasma samples paired with tumor tissue. Combining methylation and nucleosome scores into a single combined score using logistic regression did not significantly improve cluster predictability (AUC combined = 0.85) compared to the methylation score alone (AUC = 0.87).

### cfDNA methylation changes are predictive of BVZ response outcome

We next assessed methylation changes in the 40 pre- and on-treatment plasma samples in relation to survival with BVZ. We applied targeted bisulfite sequencing using our in-house developed protocol to reliably assess the methylation status of low concentrations of fragmented cfDNA in plasma. This protocol involved a SeqCap Epi capture assay containing customized capture probes, which were designed to specifically target CpGs that are unmethylated in plasma of healthy subjects and that are hypermethylated in cancer. We performed these analyses for methylation changes, but not CNA subtypes as the latter are unlikely to dynamically change during treatment. Since treatment with BVZ may reduce tumor size and therefore also the amount of cfDNA that is released in the patient blood, the methylation levels of the on-treatment samples were adjusted to accurately identify differentially methylated regions. Figure 3b demonstrates that the tumor content of most samples indeed decreases after treatment with BVZ. Therefore, the methylation values of *n* = 40 AC-ANGIOPREDICT on-treatment samples were normalized with respect to the tumor content of each plasma sample.

A methylation score was established based on the average methylation levels within each CRC specific probe region and by comparing pre-treatment samples with the methylation levels of healthy individuals. We applied the random forest of the pre-treatment samples to generate methylation scores of the normalized on-treatment samples using the random forest probability, ranging from 0 (healthy) to 1 (tumorigenic), (Fig. 3c). The methylation score of most patient samples decreased following treatment with BVZ, indicating that specific regions undergo hypomethylation during therapy.

Methylation score differences were assessed during treatment to demonstrate their potential in predicting BVZ outcome in the AC-ANGIOPREDICT cohort. Methylation score difference was calculated before and after treatment with BVZ and survival analysis was performed using multivariate cox regression correcting for clinical covariates (e.g. gender, age and TNM-stage). Samples were clustered based on the median of methylation score decreases after treatment (-0.037), with samples above this median categorized into “Small methylation decrease” samples, and samples below this median as “Large methylation decrease” samples. Large methylation score decreases contributed significantly to overall survival (Fig. 3d, *p*-value = 0.0137). In contrast, samples with a small methylation score decrease were characterized by a shorter median OS. Next, methylation score differences were assessed to see if they influenced PFS. The PFS of “Large methylation decrease” samples was initially improved compared to “Small methylation decrease” samples, but no correlation (*p*-value = 0.58) was determined (Fig. 3e). As expected, since we normalized methylation values for tumor fraction, we found no significant tumor content differences in the methylation categories, either before (*p*-value = 0.83) or after (*p*-value = 0.96) treatment with BVZ. Overall, this demonstrates that hypomethylation of CRC specific regions upon BVZ correlates with improved OS in mCRC.

## Discussion

Analysis of cfDNA in plasma is an emerging technique having a wide range of applications in the oncology setting^29^. In particular, the detection of genetic and non-genetic aberrations in cfDNA, including CNA, nucleosome scores and methylation profiles, has shown significant utility in the context of cancer diagnosis, prognosis and treatment monitoring^13,15,20,30,31^. In this study, we show that analysis of cfDNA may be used to predict outcome to BVZ in mCRC patients. Employing LC-WGS on blood and matched tumor samples from 74 mCRC patients participating in the AC-ANGIOPREDICT trial, we have developed the first prototype liquid biopsy assay for subtyping mCRC patients based on CNA profiles extracted from plasma-derived cfDNA. Moreover, we have shown how LC-WGS data can also be used for nucleosome analysis, which may represent a reliable test for malignancy, although not all differences between subtypes were significant. Finally, using *n* = 122 samples from 61 mCRC patients, we evinced significant associations between methylation changes and response to BVZ treatment.

To date, cfDNA derived CNA and CIN have been used to investigate response to treatment in in metastatic disease. For example, Weiss et al. ^32^ showed that changes in cfDNA copy number instability (CNI) score could predict standard of care chemotherapy response in metastatic cancer. Here, we firstly assessed mCRC cfDNA samples for the ability to detect CNAs, and showed that CIN patterns from cfDNA reflected those from matched tumor samples, thus confirming the potential of plasma analysis for mCRC CIN subtyping. Nevertheless, optimization of our bioinformatics pipeline was required, given the typically low tumor content in plasma^33^, which weakens segmentation of copy number events and potentially results in cluster misclassifications Thus, we incorporated a tumor content scaling step involving matching tumor tissue as reference. Most misclassifications after scaling, occurred between clusters 2 and 3, as these are similar by definition. However, as patients with high CNA levels benefit from similar clinical management, misclassifications between cluster 2 and 3 do not represent a limitation for the purpose of this study. Rather, the emphasis is on misclassification of cluster 2&3 samples into cluster 1, as patients in the latter group have worse survival compared to patients with CNA levels when treated with BVZ. With tumor samples as a reference, our method provided a 92.3% CIN classification accuracy of plasma samples, with just 7.7% of cluster 2 samples misclassified into cluster 1. The low misclassification rate thus establishes our assay prototype as a potential negative predictor of BVZ response in cluster 1 patients, allowing early stratification of patients who may gain benefit from other treatments aside from BVZ in the clinic. Overall, sensitivity of the CIN detection pipeline may be improved by deeper sequencing of samples having low tumor fraction. Moreover, the current classifier is trained using tumor tissue samples^12^ and not cfDNA, which does not account for cell type differences present in liquid biopsies. In the future development of a cfDNA trained classifier is mandated once sufficient plasma samples become available. Combination of multiple samples from different cohorts represents another strategy to improve the accuracy of cluster classification by reducing possible biases that may arise during sample collection, or between different sequencing batches.

Interestingly, we once again observed that patients (i.e. those from the AC-ANGIOPREDICT trial) with tumors classified as cluster 2 and 3 had improved OS and PFS, thus validating previous findings from our retrospective study^12^. Specifically, in our previous analyses we showed that cluster 2 and 3 patients who received BVZ combination therapy were characterized by an extended of 402 and 301 days respectively, when compared to those patients who received chemotherapy alone (253 and 216 days, *p* = 0.0098 and 0.0305 respectively). Importantly, in the current study we observed that the combination BVZ survival benefit in the CIN-high cluster (Cluster 2 with 442 days and *p* = 0.0213, Cluster 3 with 382 days and *p* = 0.0317 versus Cluster 1 with 224 days) was also reflected in the matched plasma samples (Cluster 2 with 637 days and *p* = 0.0118, Cluster 3 with 380 days and *p* = 0.0148 versus Cluster 1 with 224 days). Thus, although not all plasma samples were assigned to the same cluster as their matched tumor, cluster definition confirmed that CIN-high mCRC patients manifested improved survival compared to patients having CIN-low tumors. Among plasma samples falling into cluster 1, 57% showed no response to chemotherapy + BVZ. However, this may be underestimated due to the small number of samples (*n* = 11) which classified into cluster 1. Future studies with additional samples are mandated to confirm these findings. Overall, these results suggest that study CIN in cfDNA is a useful tool for predicting outcome and response to BVZ combination therapy in mCRC patients. While the biology underpinning the observed CIN linked response as yet unknown, in a previous study we showed that loss of the subcentromeric region of chromosome 18, 18q11.2-q12.1, predicts extended PFS in patients receiving combination BVZ^11^. One gene found on the 18q arm is SMAD4, a tumour suppressor responsible for controlling colon cell expression of VEGF and TGFβ^11,34^. Moreover, inactivation of SMAD4 by loss of the 18q chromosome arm increases expression of VEGF in colon cancer cells^34^. Thus, it is possible that increased expression of VEGF in tumors with an 18q loss results in a improved anti-angiogenic response to BVZ. Nevertheless, further work is required to fully elucidate the underlying biological mechanisms that drive the CIN-related response to BVZ. In this context, integrative multi-omic studies that combine gene expression, whole exome sequencing, single cell and spatial analyses are mandated to uncover these mechanisms. Future studies should also address issues related to low tumor content in plasma-derived cfDNA. An optimized and universal scaling method could be a powerful tool, especially in the clinical context, where patient tumor samples might not be available, and with the aim to progress towards less invasive prognostic methods^35^. Overall, while gaining access to large numbers of liquid biopsy samples is challenging, validation of our findings in extended matched plasma and tumor sample cohorts is now mandated.

As we had previously demonstrated that read-outs of CIN and NF when derived from the same cfDNA LC-WGS data, acted complementarily to detect invasive ovarian tumors^21^, we next applied a similar approach to investigate whether NFs could also be used to distinguish mCRC clusters. Having established a nucleosome score that reflected the degree of nucleosome position deviation for each mCRC plasma sample, we observed a significant difference in the nucleosome scores between cluster 1 and 2 samples (within the AC-ANGIOPREDICT cohort), although not with cluster 3 since these samples displayed a heterogeneous nucleosome score profile. We observed that nucleosomes scores correlated strongly with tumor fractions in each cfDNA samples, as determined by WisecondorX, indicating that nucleosome positioning using LC-WGS may serve as a proxy for CIN and CIN subtyping. Cristiano et al. ^14^ previously demonstrated the power of combining nucleosome footprinting with copy number changes in patients with mCRC. However, the samples in this study were processed at a coverage of 1–2×, while the samples in AC-Angiopredict had a coverage of only 0.2–0.5×, which could explain the deviating nucleosome footprints of some cluster 3 samples. NF in combination with CIN profiling of cfDNA may be a powerful method to determine malignancy and cluster identification in low tumor content cfDNA.

Previously, we have shown that tumor hypoxia, a known inducer of EMT, can directly impair the activity of ten-eleven translocation (TET) DNA demethylases by reducing the availability of oxygen, an essential cofactor of TET enzymes^24^. Conversely, BVZ treatment has been reported to normalize the tumor vasculature, thereby reducing tumor hypoxia levels and thus increasing oxygen levels^6^. Based on observations in mouse tumor models, this increased availability of oxygen can increase DNA demethylation activity by the TETs and lead to a reduction in DNA methylation. These data provided a rationale to explore methylation dynamics with respect to BVZ outcome. In this context we assessed whether different CIN clusters displayed distinct methylation patterns. Overall, we observed significantly lower baseline methylation scores in cluster 1 samples compared to cluster 2 and 3, suggesting that these scores could indeed discern between high and low CIN samples. We hypothesize that the low baseline methylation levels in cluster 1 tumours are a function of the underlying biology of tumours. For instance several cluster 1 tumors are characterized by microsatellite instability (MSI), a hypermutable phenotype caused by a decrease in DNA mismatch repair activity, which may lead to lower baseline methylation levels. Overall, methylation scores were superior to nucleosome scores in differentiating cluster 1 from cluster 2 and 3 samples (AUC = 0.87 compared to 0.75). Next we analysed cfDNA methylation scores, following normalization for tumor fraction, before and following treatment with BVZ. We observed that patients with a large methylation score decrease were associated with significantly longer OS, compared with patients manifesting a small methylation score decrease. Overall methylation score decreases were prognostic for improved BVZ outcome. Thus, our hypothesis that BVZ exerts its therapeutic effects by normalizing the tumor vasculature has been confirmed.

Limitations of the current study include the relatively small available sample size and matching tumour samples not being available for the validation cohort (UMM). Notwithstanding these limitations, we nevertheless verified the protocol for identifying cfDNA CIN clusters from plasma (using the UMM cohort) and demonstrated predictive potential of the biomarker using the available sample cohort, shown in Fig. 1f, g.

In conclusion, having the ability to derive clinically relevant information from liquid biopsies presents a step forward towards the shift to alternative, minimally invasive techniques to confirm diagnosis and direct treatment in the setting of mCRC. The clinical utility of liquid biopsy screening for CNA in mCRC has the potential to facilitate CIN stratification of mCRC patients into BVZ responders and non-responders, thus optimizing patient treatment and improving overall patient management. Given the significant side effects related to the administration of BVZ, an informed patient selection process for BVZ treatment would potentially improve quality of life for non-responders and reduce healthcare costs^36^. Here, we have shown how cfDNA may be analysed to predict outcome to BVZ in a cohort of mCRC patients. Moreover, detection of CNAs and methylation profiles allowed the stratification of samples into CIN clusters and provide patient response data. Plasma liquid biopsies hold promise for improved precision treatment and patient management in the mCRC setting.

## Methods

### Sample collection

#### AC-ANGIOPREDICT cohort

Serial tumor and blood samples (stored at −80 °C) were prospectively collected (between April 2013 and February 2015, with a median follow up time of 22 months) from *N* = 74 mCRC patients participating in the AC-ANGIOPREDICT phase II clinical trial (NCT01822444, trail ended in Jan 2017)^37^. Of the 74 patients, 52 matching tumor-tissue plasma samples were available for CIN classification, concordance testing and nucleosome foot printing analysis. For 61 of the 74 AC-ANGIOPREDICT mCRC patients, plasma samples before and after treatment (week 6) with BVZ were collected from patients, resulting in a total of 122 samples that were used for targeted methylation sequencing. Tumor and blood samples were collected prior to administration of first line SOC chemotherapy + BVZ. Treatment response was assessed by standard radiologic imaging (CT). Tumor samples were formalin fixed paraffin embedded (FFPE), and all samples underwent central pathology review by the ANGIOPREDICT consortium pathologist, and nucleotide (DNA/RNA) extraction from 20 µm sections having >40% tumor content was performed, according to protocols developed during the ANGIOPREDICT project^12^. All patients enrolled on the AC-ANGIOPREDICT trial provided written informed consent. Clinical data is shown in Table 4. All patients enrolled on the AC-ANGIOPREDICT trial (NCT01822444) were provided written informed consent in accordance with the standards proposed by the Declaration of Helsinki. The trail protocol was approved by Cancer Trials Ireland scientific management committee (formally ICORG) in 2012. The following hospital research ethics committees (REC) in Ireland approved to study: Beaumount Hospital REC, Dublin, Adelaide and Meath Hospital REC, Dublin, St James’s Hospital REC, Dublin, St. Vincent’s University Hospital REC, Dublin, Bon Secours REC, Cork, University Hospital Waterford REC. German Ethics boards: University Hospital Mannheim REC, University Hospital Mainz REC, Klinikum Ludwigsburg REC, and Oncology Centre Speyer REC.

**Table 4:** Summary of clinical information for mCRC patients from the AC-ANGIOPREDICT (AC-ANGIOPREDICT) cohort (n = 74 plasma samples, NCT01822444) and University of Mannheim (UMM) cohort (n = 24 plasma samples)

Clinical Info				
	AC-ANGIOPREDICT		UMM	
	*n* = 74	%	*n* = 24	%
*Gender*				
Female	26	37.8	5	20.8
Male	48	62.2	19	79.2
*Age (years)*				
>65	17	23	8	33.3
≤65	55	74.3	16	66.7
Missing values	2	2.7	0	0
*T-classification*				
1	1	1.4	0	0
2	4	5.4	0	0
3	29	39.2	0	0
4	26	35.1	0	0
Missing values	14	18.9	25	100
*N-classification*				
0	14	18.9	0	0
1	23	31.1	0	0
2	17	23	0	0
Missing values	20	27	25	100
*M-classification*				
0	11	14.9	0	0
1	58	78.4	0	0
Missing values	5	6.7	20	100
*KRAS*				
wt	17	23	10	41.6
mut	56	75.7	14	58.4
Missing values	1	1.3	0	0
*BVZ*				
Yes	74	100	17	70.8
No	0	0	7	29.2
*Backbone*				
FP-OX	46	62.2	9	37.5
FP-IRI	22	29.7	14	58.3
FP-OX-IRI	0	0	1	4.2
Missing values	6	8.1	0	0
Total	74	100	24	100

*SOC* standard-of-care, *wt* wild-type, *mut* mutated, *BVZ* bevacizumab, *FP* fluoropyrimidin, *IRI* irinotecan, *OX* oxaliplatin

#### University of Mannheim cohort (UMM)

Serial blood samples were also collected from *n* = 24 mCRC patients treated at University Medical Center Mannheim (Heidelberg University, Germany) and provided to the current study by the Mannheim Liquid Biopsy Unit—MaLiBU biobank (stored at −80 °C). These samples were used as a validation cohort for CIN clustering in plasma cfDNA. Samples from patients undergoing palliative chemotherapy ± BVZ between 2015 and 2019 were selected. Blood samples were obtained before start of treatment and/or parallel to radiologic assessment of therapy response. For each patient, longitudinal samples were collected before treatment and while on BVZ or chemotherapy treatment. Written informed consent was obtained from all patients, and the biobank was approved by the local ethics board (Ethikkommission II, Medical Faculty Mannheim, Heidelberg University, identifier 2013-640N-MA) in accordance with the standards proposed by the Declaration of Helsinki. Clinical information is shown in Table 4.

### Next generation sequencing

#### Low-coverage whole-genome sequencing (LC-WGS)

Plasma-derived cfDNA from the AC-ANGIOPREDICT (*n* = 74) was extracted via a 2-step centrifugation procedure as previously described^20^. Tumor DNA from biopsies was extracted using the QIAamp circulating nucleic acid kit (Qiagen, UK). Shot-gun whole genome libraries were prepared using the KAPA library preparation kit (KAPA Biosystems). For library enrichment, 5–15 cycles of PCR with intermediate assessment steps were used to ensure low adapter dimer content and high library yield. DNA libraries constructed from AC-ANGIOPREDICT plasma and tumor samples were sequenced using LC-WGS up to a sequencing depth of 0.1–0.2× coverage on an Illumina HiSeq 4000 (50nts read length), as described previously^15^. (Further bioinformatics analysis methods can be found in Supplementary Methods).

#### Targeted bisulphite sequencing

2–40 ng of input cfDNA for 61 patients were subjected to bisulphite conversion and the Accel-NGS kit (Swift BioSciences) was used to generate functional double-stranded, bisulphite-converted, indexed libraries. Subsequently, a subset of the genome was captured by a pool of 25,399 customized capture probes (SeqCap Epi, Roche, Basel, Switzerland). Particularly, a published Illumina 450k methylation array dataset on plasma-derived cfDNA samples of 656 healthy individuals (GSE40279) was used to select 44,341 unmethylated target CpGs (mean average methylation beta-value < 0.03 across all samples) [18]. In total, 25,399 SeqCapEpi capture probes (length range: 59–1037 bp) were designed from the 44,341 target CpGs. Captured libraries were then sequenced on an Illumina HiSeq4000 (paired-end 2*150 bp reads). After quality control of the sequencing results from the 122 cfDNA samples (61 before treatment with BVZ, 61 after treatment with BVZ), 23 samples were excluded from further analysis: one post-treatment sample due to a bisulphite conversion rate below 95% and 22 samples (18 pre-treatment, 4 post-treatment) because the mean coverage of the captured regions was below 10×. Thus, further analysis was performed on the remaining 99 samples. In addition, a reference set of 41 female healthy individuals (approved by the Ethics Committee UZ/KU Leuven—S64035) was used for the methylation analysis (median age of 44 years with interquartile range: 25–63).

#### Sample size and statistical analysis

In our previous paper^12^ which leveraged a cohort of 392 mCRC patients where we found that circa 10% of all samples represent CIN subtype 1 using low-coverage whole-genome sequencing. Thus for the current study, to determine the minimal sample size of the respective cohorts ensuring at least one sample with CIN subtype 1, we defined a binomial distribution with the following proportions for cluster 1 (0.1) and cluster 2 and 3 (0.9). For the CIN biomarker, the minimal sample size was calculated with *α* = 0.05. Hence it was calculated that a minimum of 29 patients was required to obtain 95% confidence in finding at least one patient in cluster 1. The AC-ANGIOPREDICT cohort contained 74 patients. For the UMM cohort we were unable to retrieve more than 24 patients with informed consent. The provided sample size corresponds with a confidence level of 92% of containing at least one cluster 1 sample.

Boxplots were plotted using ggplot2 (version 3.6.3)^38^. Upper and lower edges correspond with the first and third quartile, respectively. Upper and lower whiskers of each boxplot correspond with the closest observation of 1·5 times the interquartile range with respect to each edge. For clarity, data points are plotted on top of the boxplots with random variation in the horizontal direction to avoid overlap. Mann-Whitney tests were used to compare CIN subtypes. Correlations between metrics were calculated using Spearman’s correlation coefficients. All data was analysed in R version 3.6.3^39^.

### Reporting summary

Further information on research design is available in the Nature Research Reporting Summary linked to this article.

### Supplementary information

Supplementary Information

REPORTING SUMMARY

## Data Availability

The sequencing data are deposited at the European Genome—Phenome Archive (https://ega-archive.org/) under accession code EGAS50000000131 and are available available from the corresponding author on reasonable request.

## Code availability

The software tools and bioinformatics pipelines used for data analysis have been reported in the Methods section.
