# Supplementary material for: Analysis of cell free DNA to predict outcome to bevacizumab therapy in colorectal cancer patients
Source: NPJ Genom Med. 2024 May 29;9:33. doi: 10.1038/s41525-024-00415-x (PMC11137102; doi:10.1038/s41525-024-00415-x)
Supplement: Supplementary file 1 — Supplementary Information [file 41525_2024_415_MOESM1_ESM.pdf]

SUPPLEMENTARY FIGURES

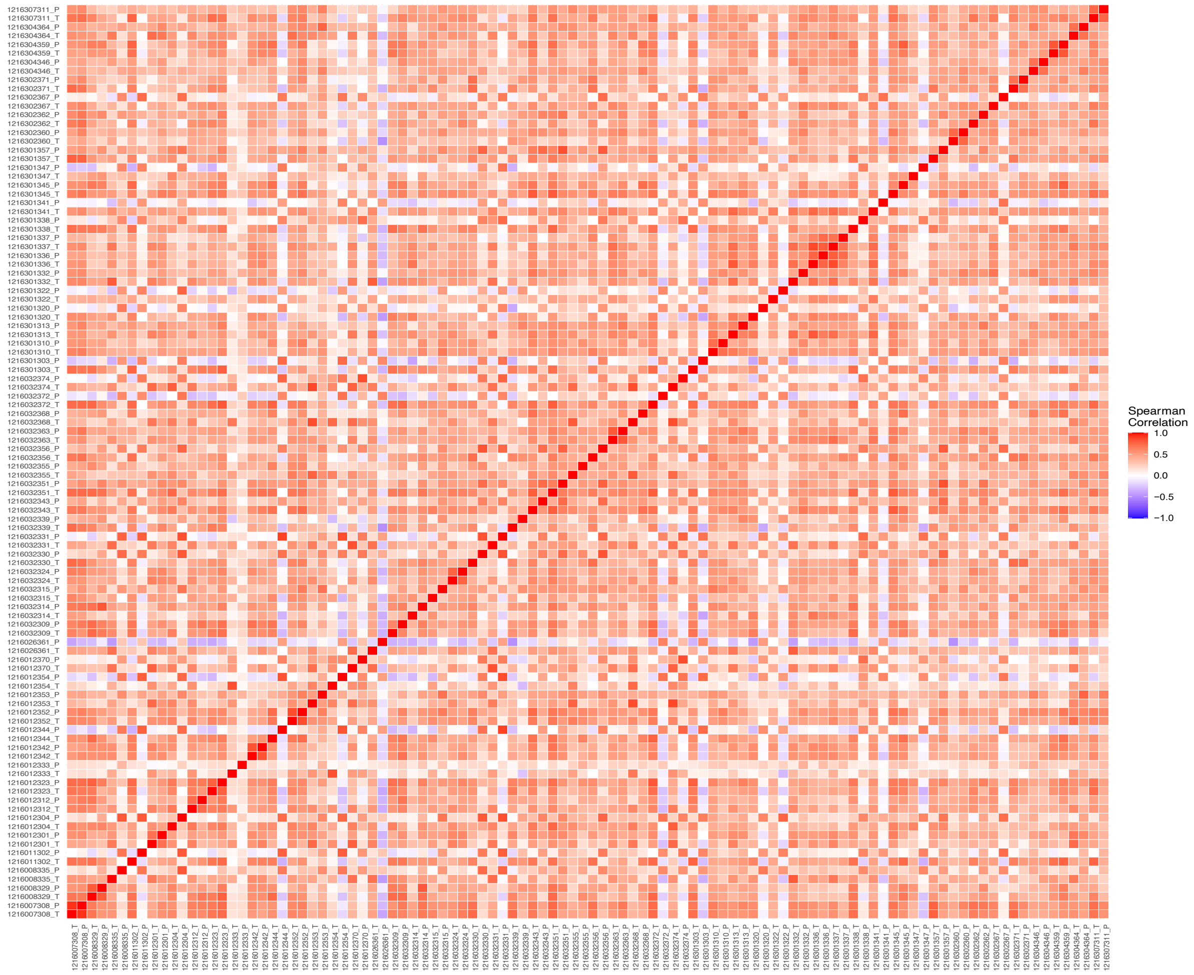

Supplementary Figure 1. Spearman correlation analysis of the LogR values of both tumor tissue and corresponding plasma samples in the AC-ANGIOPREDICT cohort. Color-coded representations are based on Spearman's rank correlation coefficients.

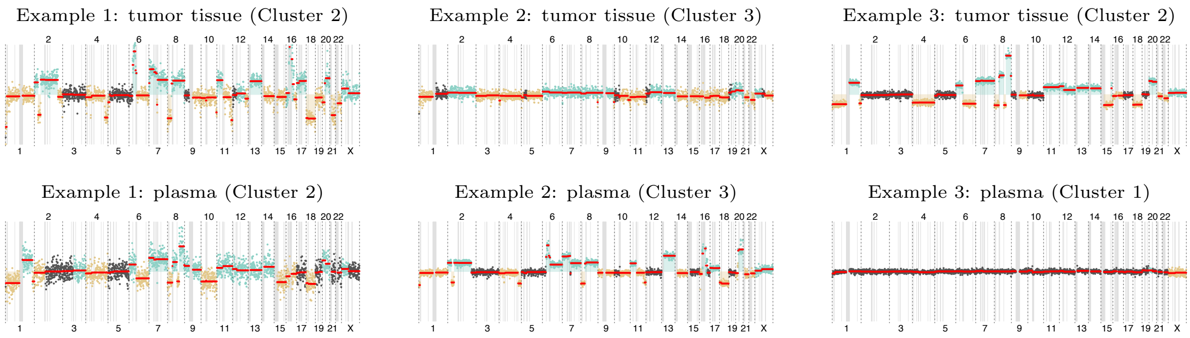

Supplementary Figure 2. Comparison of paired tumor tissue and plasma copy number profiles from the AC-ANGIOPREDICT cohort. Genomic representation profiles were obtained from fresh frozen tumor tissue (top) and matching plasma samples (bottom). Example 1 and 2 illustrate matching classifications between tumor tissue and plasma for cluster 2 and cluster 3, respectively. Example 3 illustrates an incorrect classification (cluster 2 in tumor tissue, cluster 1 in plasma), owing to low tumor fraction in plasma. Designated clusters for each sample were determined by processing and scaling of the profiles with WisecondorX. Significant copy number amplifications and deletions are coloured using light-blue and orange dots, respectively. Insignificant copy number changes are illustrated using black dots. The segmented profile is coloured using a red line.

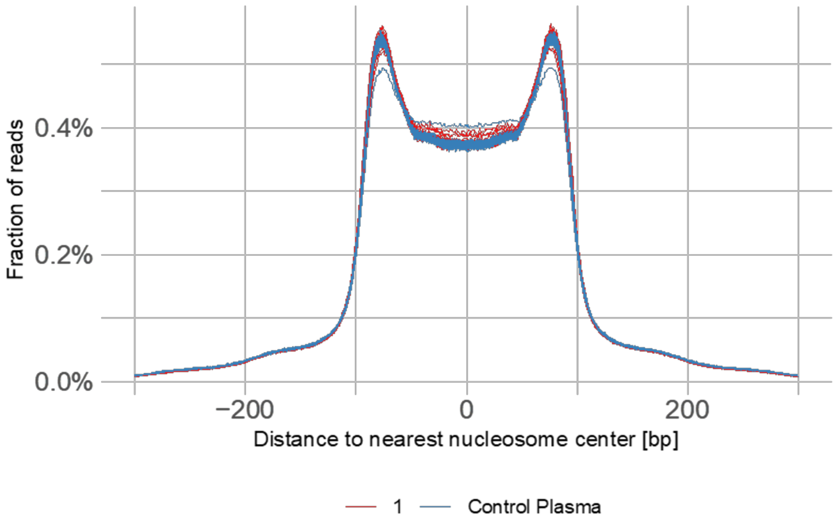

Supplementary Figure 3: Nucleosome profile of Cluster 1 samples compared to healthy controls. An M-shaped profile is found when a genome-wide distribution of the distances between the start of each read and the center of the nearest nucleosome of Cluster 1 samples is plotted. AC-ANGIOPREDICT mCRC cluster 1 plasma samples are shown in blue lines while healthy control samples are shown in red lines.

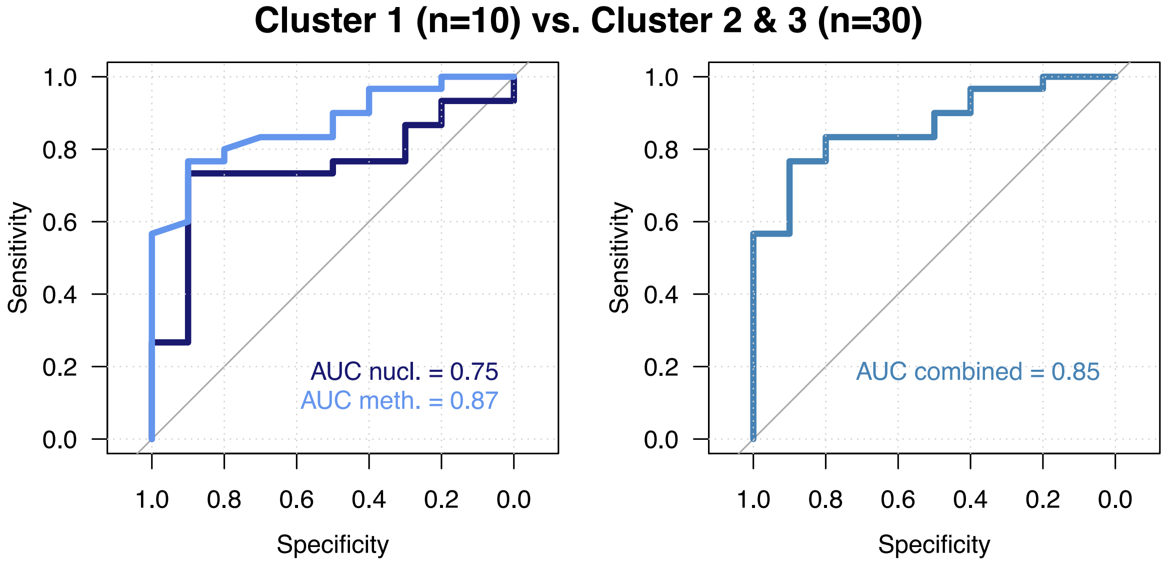

Supplementary Figure 4: ROC curve analysis of mCRC clusters. Left panel: ROC curves of cluster 1 versus cluster 2 and 3 samples. Individual curves for each metric: nucleosome score (nucl.) and methylation score (meth.). Right panel: combination of the nucleosome and methylation score with an optimism-corrected AUC value using logistic regression.

Supplementary Methods

Bioinformatics pipeline

Mapping and copy number calling of LC-WGS cfDNA.

Raw sequencing reads of 52 tumor tissue samples and 98 plasma samples were processed with BWA (version 0.7.15) [1] using the hg19 reference genome. Duplicated and low-quality reads were filtered using Picard (version 1.141) [2]. The QDNAseq software package (version 1.22) [3] was used to create bins of 100kb and to transform the resulting number of reads per 100 Kb window into log R-values. ASCAT (version 2.0.1) [4] was used to segment the reads processed by QDNAseq and to determine tumor fraction and overall ploidy of the tumor tissue and plasma samples. Finally, significant copy number alterations (CNAs) across all samples were determined using GISTIC (version 2.0) [5], using settings as described previously [6]. In addition to ASCAT, we also performed copy number segmentation using ichorCNA (version 0.3.2) [7], a CNV software developed in the context of LC-WGS sequencing samples. Low tumor fraction threshold settings were used with the following modified flags: --maxCN 3 --estimateScPrevalence FALSE --scStates "c()" --chrs "c(1:22)" --chrTrain "c(1:22)". In parallel, segmentation was also performed with WisecondorX (version 1.2.4) [8], a segmentation tool that includes an improved normalization procedure for processing of plasma samples with low-tumor content by using a reference set of 100 healthy control plasma samples (approved by the Ethics Committee UZ/KU Leuven – S64035).

Concordance between the LC-WGS copy numbers of tumor tissue and matching plasma samples.

We compared the segmented logR values of the matching tumor tissue and plasma samples by generating copy number profiles using ASCAT (version 2.0.1), ichorCNA (version 0.3.2) and WisecondorX (version 1.2.4). In total, we obtained matching data for 52 of the 74 patients. Since the CNAs were underrepresented in plasma samples due to dilution of the circulating tumor DNA, we implemented a gradual scaling of the logR values (i.e., readout value for CNV profiles) using, i) the difference between tumor fraction in tumor tissue with respect to the matching plasma sample, and ii) the rank of each logR value in the plasma sample so that the largest values (likely CNA) are amplified the most, while smallest values (possibly noise) are amplified the least. ASCAT does not provide a tumor fraction estimate for samples with a low tumor content (<20%), therefore the tumor estimation from ichorCNA was used instead. For WisecondorX, the average Z-score of the segmented values was used as an approximation of the tumor fraction.

Statistical analysis of LC-WGS samples.

CNA clusters were determined using a random forest approach and 10-fold cross-validation on the original mCRC dataset from Smeets *et al.* [6], encompassing 472 mCRC tumor samples. Briefly, random forest classification was performed using the R-package "randomForest" (version 4.6-14) [9]. The cfDNA SCNA classifier categorized each of the plasma and tumor samples to one of our three previously described mCRC CIN clusters. To show the benefit of BVZ in CNA medium-high clusters, multivariate survival analysis between and within the different clusters was performed using a Cox regression analysis with the R-package "survminer" (version 0.4.9) [10]. TNM staging (a classification for cancer staging using tumour site and size (T), involvement of lymph nodes (N)

and presence of metastasis (M)) and age were used as numerical factors while gender, cluster membership or CNA were considered as categorical variables.

Calculation of nucleosome scores from LC-WGS sequencing data.

The genome-wide deviation of the nucleosome positioning was quantified by calculating a nucleosome score based on the cfDNA fragmentation patterns from the baseline LC-WGS sequencing data. Briefly, the distances between the start of a mapped sequencing read (i.e., the boundary of a circulating cfDNA fragment) and the nearest nucleosome center from a publicly available reference list of nucleosome positions in healthy individuals [11] were calculated. Multinomial modelling was performed using a positive reference set of four mCRC plasma cases and a negative reference set of healthy individuals, as described previously [12]. The distribution of these distances displays a typical M-shaped profile. Using the deviation in the M-shaped profiles between the positive reference set and the negative reference set, a nucleosome score reflecting a numeric read out of this deviation between 0 and 1 was calculated for each of the plasma samples of mCRC patients using Bayesian hierarchical modeling in R, version 3.6.3. Values around 0 correspond to reference healthy profiles while values around 1 correspond to tumor profiles.

Mapping of targeted bisulfite-sequencing cfDNA.

The sequencing reads were trimmed using TrimGalore (version 0.4.1) [13]. The trimmed FASTQ files were mapped on a bisulfite-converted human genome (GRCh37) using Bismark [14]. Coverage files were extracted using Bismark's methylation extractor which quantifies the degree of methylation

for each CpG position. From the 122 starting samples, we excluded 23 samples (18 pre-treatment, 5 post-treatment) due to low coverage and/or low bisulfite conversion efficiency. The sequencing reads of the remaining 99 cohort samples were subsequently filtered based on their full overlap with CRC tumor specific probe regions. These CRC tumor specific probe regions were determined by selecting hypermethylated CpGs in publicly available methylation array data from CRC samples (TCGA CRC primary tumor, 313 samples) with respect to a healthy control plasma sample dataset (GSE40279, 656 samples) [15] using the TCGAbiolinks package (version 2.14.1) [16]. In total, we retrieved 31,918 hypermethylated CpGs in CRC compared to the controls, of which 3,894 CpGs overlapped with the pool of 28,025 customized capture probes, resulting in the selection of 2,107 unique CRC-specific capture probes for further analysis. Downstream analysis to calculate the methylation score of cfDNA in each sample was performed in R (version 3.6.3). Random forest modelling using the R-package “randomForest” (version 4.6-14) [9] with 10-fold cross-validation generated methylation scores in cfDNA collected from the AC-ANGIOPREDICT plasma samples before treatment (n=40) and healthy controls (n=41) using the random forest probability, ranging from 0 (no tumor content) to 1 (only tumor content).

Estimation of tumor fraction of bisulfite sequencing samples.

Since treatment with BVZ may reduce the size of a tumor and therefore also the amount of circulating tumor DNA (ctDNA) that is released in the patient blood, the methylation levels were adjusted to accurately identify differentially methylated regions in the samples before and after treatment with BVZ. We therefore adjusted our cfDNA protocol and normalized the methylation values by calculating the tumor content of each sample using the above described ichorCNA

bioinformatics tool [7]. Note that the capture protocol will relatively enrich the number of reads in the probe regions with respect to other genome regions. These captured regions may erroneously be identified as copy number amplifications; therefore, all reads were filtered based on their overlap with the target regions and only non-target reads were kept for calculation of the tumor content.

1 Li, H. & Durbin, R. Fast and accurate long-read alignment with Burrows-Wheeler transform. *Bioinformatics* **26**, 589-595, doi:10.1093/bioinformatics/btp698 (2010).

2 Picard toolkit (Broad Institute, GitHub repository, 2019).

3 Scheinin, I. *et al.* DNA copy number analysis of fresh and formalin-fixed specimens by shallow whole-genome sequencing with identification and exclusion of problematic regions in the genome assembly. *Genome Res* **24**, 2022-2032, doi:10.1101/gr.175141.114 (2014).

4 Van Loo, P. *et al.* Allele-specific copy number analysis of tumors. *Proc Natl Acad Sci U S A* **107**, 16910-16915, doi:10.1073/pnas.1009843107 (2010).

5 Mermel, C. H. *et al.* GISTIC2.0 facilitates sensitive and confident localization of the targets of focal somatic copy-number alteration in human cancers. *Genome Biol* **12**, R41, doi:10.1186/gb-2011-12-4-r41 (2011).

6 Smeets, D. *et al.* Copy number load predicts outcome of metastatic colorectal cancer patients receiving bevacizumab combination therapy. *Nat Commun* **9**, 4112, doi:10.1038/s41467-018-06567-6 (2018).

7 Adalsteinsson, V. A. *et al.* Scalable whole-exome sequencing of cell-free DNA reveals high concordance with metastatic tumors. *Nat Commun* **8**, 1324, doi:10.1038/s41467-017-00965-y (2017).

8 Raman, L., Dheedene, A., De Smet, M., Van Dorpe, J. & Menten, B. WisecondorX: improved copy number detection for routine shallow whole-genome sequencing. *Nucleic Acids Res* **47**, 1605-1614, doi:10.1093/nar/gky1263 (2019).

9 Liam, A. & Wiener, M. Classification and Regression by randomForest. *R News* **2**, 18-22 (2002).

10 survminer: Drawing Survival Curves using 'ggplot2' (2020).

11 Snyder, M. W., Kircher, M., Hill, A. J., Daza, R. M. & Shendure, J. Cell-free DNA Comprises an In Vivo Nucleosome Footprint that Informs Its Tissues-Of-Origin. *Cell* **164**, 57-68, doi:10.1016/j.cell.2015.11.050 (2016).

12 Vanderstichele, A. *et al.* Nucleosome footprinting in plasma cell-free DNA for the pre-surgical diagnosis of ovarian cancer. *NPJ Genom Med* **7**, 30, doi:10.1038/s41525-022-00300-5 (2022).
13 Krueger, F. "Trim galore." A wrapper tool around Cutadapt and FastQC to consistently apply quality and adapter trimming to FastQ files. (2015).
14 Krueger, F. & Andrews, S. R. Bismark: a flexible aligner and methylation caller for Bisulfite-Seq applications. *Bioinformatics* **27**, 1571-1572, doi:10.1093/bioinformatics/btr167 (2011).
15 Hannum, G. *et al.* Genome-wide methylation profiles reveal quantitative views of human aging rates. *Mol Cell* **49**, 359-367, doi:10.1016/j.molcel.2012.10.016 (2013).
16 Colaprico, A. *et al.* TCGAbiolinks: an R/Bioconductor package for integrative analysis of TCGA data. *Nucleic Acids Res* **44**, e71, doi:10.1093/nar/gkv1507 (2016).
